# Sustainability of evidence-based healthcare: research agenda, methodological advances, and infrastructure support

**DOI:** 10.1186/s13012-015-0274-5

**Published:** 2015-06-11

**Authors:** Enola Proctor, Douglas Luke, Annaliese Calhoun, Curtis McMillen, Ross Brownson, Stacey McCrary, Margaret Padek

**Affiliations:** George Warren Brown School of Social Work, Washington University in St. Louis, One Brookings Drive, Campus, Box 1196, St. Louis, MO USA; Bridgespan Group, 2 Copley Pl #3700b, Boston, MA USA; School of Social Service Administration, The University of Chicago, 969 E. 60th Street, Chicago, IL USA

**Keywords:** Sustainability, Implementation research

## Abstract

**Background:**

Little is known about how well or under what conditions health innovations are sustained and their gains maintained once they are put into practice. Implementation science typically focuses on uptake by early adopters of one healthcare innovation at a time. The later-stage challenges of scaling up and sustaining evidence-supported interventions receive too little attention. This project identifies the challenges associated with sustainability research and generates recommendations for accelerating and strengthening this work.

**Methods:**

A multi-method, multi-stage approach, was used: (1) identifying and recruiting experts in sustainability as participants, (2) conducting research on sustainability using concept mapping, (3) action planning during an intensive working conference of sustainability experts to expand the concept mapping quantitative results, and (4) consolidating results into a set of recommendations for research, methodological advances, and infrastructure building to advance understanding of sustainability. Participants comprised researchers, funders, and leaders in health, mental health, and public health with shared interest in the sustainability of evidence-based health care.

**Results:**

Prompted to identify important issues for sustainability research, participants generated 91 distinct statements, for which a concept mapping process produced 11 conceptually distinct clusters. During the conference, participants built upon the concept mapping clusters to generate recommendations for sustainability research. The recommendations fell into three domains: (1) pursue high priority research questions as a unified agenda on sustainability; (2) advance methods for sustainability research; (3) advance infrastructure to support sustainability research.

**Conclusions:**

Implementation science needs to pursue later-stage translation research questions required for population impact. Priorities include conceptual consistency and operational clarity for measuring sustainability, developing evidence about the value of sustaining interventions over time, identifying correlates of sustainability along with strategies for sustaining evidence-supported interventions, advancing the theoretical base and research designs for sustainability research, and advancing the workforce capacity, research culture, and funding mechanisms for this important work.

## Background

This paper addresses one of the most significant translational research problems of our time: in spite of rapid advances in evidence-based medicine, we know very little about how well or under what conditions health innovations are sustained and their gains maintained once they are put into practice. Implementation science typically focuses on initial uptake by early adopters of one healthcare innovation at a time. Later-stage challenges of scaling up and sustaining evidence-supported interventions have received too little attention. Consequently, Americans receive less than adequate care [1, 2]. Comparative effectiveness research (CER) can help determine optimal interventions; however, reaping a return on the nation’s CER investment requires that healthcare settings integrate and sustain effective interventions over time.

Sustainability is a key implementation outcome [3] and a priority topic in implementation science [4]. Sustained delivery of evidence-based interventions is essential to public health impact [5]. Yet sustainability remains one of the least understood and most vexing issues for implementation research, largely due to unique methodological challenges. This paper reports results of concept mapping (CM) focused on sustainability and a subsequent working conference that generated recommendations for sustainability research. Recommendations include topics nominated for a substantive research agenda on sustainability and methodological and infrastructure advances that are important for sustaining evidence-based innovations in health.

Several studies have investigated the sustainability of innovations in mental healthcare [6–8], prevention [5], schools [9], primary care [10, 11], and acute care settings [12–16]. Stirman and colleagues’ review article synthesized and derived implications from the empirical research on sustainability [17]. The literature includes published frameworks for implementing sustainability interventions [18], measurement frameworks for sustainability [19], and foundational agendas for research on sustainability [20, 21]. Reported rates of sustainability in clinical studies range from very low—in settings that are low resourced [10] or experience high staff turnover [9] to fairly high. The sparse available literature suggests that intervention adaptation, fit with context, continual financial support, training, fidelity, and leadership contribute to sustainability [6, 17, 20].

This literature reflects a myriad of methodological issues. Sustainability itself is called by different names, conceptualized differently, and measured with different metrics and observation periods. Many studies do not provide operational definitions of sustainability and most rely on naturalistic designs using retrospective self-report data [6, 17]. Sustainability rates are sometimes reported as percent of sites that sustain the practice [6] and sometimes by rate of outcome improvement [14]. Since most data collection ends when grant funding stops, information about sustainability is typically beyond the purview of effectiveness RCT’s and health services research. Thus, the event of primary interest—the sustained use of the intervention protocol tested—is typically not captured by conventional research designs.

### Project aims and rationale

We sought to identify the challenges associated with sustainability research from thought leaders in the field and to generate recommendations for strengthening this work. We used a multi-method, multi-stage approach, including CM and action planning in an intensive working conference supported by an R13 mechanism, to pursue these specific aims:

1. Identify thought leaders and stakeholders with shared investment in the sustainability of effective healthcare practices;
2. Support their collaboration in identifying the most important and most challenging issues in sustainability research; and
3. Generate a detailed agenda for studying sustainability, including recommendations for research, policy, and practice action toward sustaining effective healthcare innovations.

This work was guided by the following assumptions. First, the sustainability challenge spans all of healthcare—acute care medicine, primary care, behavioral healthcare, and public health practice and policy. Thus, we sought to achieve generalizability by focusing on sustainability across healthcare settings, service delivery sectors, diseases, and funding sources. Second, a wide range of stakeholders have investment in sustainability, including (1) researchers who develop, test, and disseminate interventions and programs in hopes of seeing them sustained in real-world care; (2) health leaders who seek a return on their provider training investments; (3) health advocates who are wary of researchers who bring new interventions to their settings only for the duration of grant funding and leave little sustainable change at study’s end; and (4) funders whose grants support basic, clinical efficacy, and service system research and want sustained public health impact from those discoveries. Our project included these perspectives. Finally, we sought to understand “de-adoption” of practices given the limited “absorptive capacity” [22] of systems and the ongoing supply of new research on effective programs and interventions [23].

## Methods

We chose methods that would bring experts together (virtually and in person), stimulate new thinking, and shape ideas built upon already published sustainability research. The project employed multiple methods across four phases, detailed below.

### Phase one: identifying and recruiting sustainability experts

To identify leaders in the field of sustainability, we searched Academic Premier, Medline, PsychINFO, and CINHL for peer-reviewed articles in the years 2000–present, using search terms such as sustain, implement, disseminate, evaluate, mental health, healthcare, systems of care, institution, public health, and develop. We also employed snowballing strategies by searching citations in published articles on sustainability. An NIH reporter search yielded 26 grants, from which we captured grant titles, project officers, and principal investigators. Finally, we contacted project officers at research funding agencies, including the AHRQ, NIH Institutes, the Robert Wood Johnson Foundation, and the Centers for Disease Control, to identify investigators with new projects not yet published or shown in NIH Reporter. From these sources, we populated nine cells: researchers, practice leaders, and research funders in each of three areas: behavioral health (*n* = 24), public health (*n* = 34), and medicine (*n* = 27). Our fully populated grid contained 94 names, which we contacted to determine their interest in participation.

### Phase two: conducting CM research on sustainability

We used CM methodology to capture thought leaders’ perspectives on key issues in sustainability research. Widely used in public health and health services research [24–27], CM is a mixed methods approach for conceptualizing a topic [28]. CM elicits data using qualitative procedures, funnels them through software (www.conceptsystems.com), analyzes data quantitatively, and yields a visual representation of data in a “map”. We chose CM because of its capacity to engage multiple and broadly dispersed participants in idea generation and produce group-level perceptions of the data. CM’s visual representation facilitates interpretation and action planning. CM involves six distinct processes: preparation, brainstorming, sorting, ranking, establishing “go zones”, and action planning. We developed the focus prompt for group brainstorming: “*In advancing a program of research on sustainability*, *an important issue is*…”

We invited the 94 experts to participate in brainstorming. An email defined CM, outlined the phases of participation, invited participation in (anonymous) brainstorming, and invited online consent. The brainstorming page was accessed 50 times, indicating up to 50 participants. In response to the focus prompt, participants generated 136 original statements. We cleaned and consolidated these statements, yielding 91 unique statements for subsequent ranking and sorting phases.

Sixty-five individuals were invited to participate in the sorting and ranking phases, with criteria including: 1) prior invitation to brainstorming (given anonymity, we did not know which individuals had participated); 2) corresponded to one of the priority roles (researchers, funders, and healthcare leaders) and healthcare arenas (physical health, mental health, and public health); 3) current involvement in sustainability work; and 4) diversity in gender, race, and level of research experience (junior and senior investigators). When individuals were research collaborators, we chose only one team member. Again, participation required online consent. In the sorting phase, participants distributed the 91 brainstormed items according to groupings and assigned labels to these groups. For example, the following statements were grouped together and labeled, “*Factors affecting sustainability*: *Understanding which variables are more important for sustainability than others*, *and understanding the reasons why strategies are/are not sustained*”. The sorting required about 60–100 min per participant.

In the rating phase, participants comparatively ranked each of the 91 statements according to two different scales: (1) its importance (1 = not at all important, 5 = extremely important) for advancing research on sustainability, and (2) its challenge for the field (1–5 ranking). While many CM exercises ask participants to rate statements according to importance and feasibility, we chose to elicit ratings of degree of challenge because we felt research should prioritize more challenging issues. The final response rate for sorting and rating was low, likely due to the complexity of the topic, the time intensity, and our inability to offer financial incentives. We received usable data from 19 people in sorting, 18 in the importance rating, and 13 in the challenge rating. The methodology yielded rich and useful data for the conference, providing a basis for participants to further develop, refine, and advance ideas.

After brainstorming and rating phases are complete, the software is used to perform cluster analysis and multidimensional scaling (MDS) which enable visual representation of the data in the form of clusters. Randomly numbered statements are configured in a “point map” with conceptually similar ideas situated in close proximity and different ideas placed further apart. The core study team (paper authors) worked systematically to identify the most useful number of clusters, considering the range of issues represented, the bridging value of each item (a score calculated by Concept Systems indicating how frequently an item was grouped with other items), the purpose and intended uses of the map, and the observed coherence of clusters at different levels. Working from an initial 9-cluster map that provided a good general fit to the data, the team finalized an 11-cluster map, labeled and ready for action planning by conference participants.

CM methodology also enables construction of go zones, which are bi-variate scatter plots based on importance ratings on the x-axis and challenge ratings on the y-axis. In a four quadrant graph, the upper right quadrant represented ideas rated as both “highly important” and “very challenging”; the lower right, highly important but less challenging; the lower left, not important and not challenging; and the upper left, not as important but challenging.

### Phase three: action planning through an in-person working conference

Building on recommendations from the 2010 AHRQ-supported conference on scale up and spread [29], we convened a two-day conference of public policy leaders, donors, practitioners, and researchers around the topic of *Sustaining the gains: Advancing Sustainability Research*, February 9–10, 2012, at Washington University in St. Louis. Sessions were designed to succinctly present a synopsis of extant sustainability research, engage participants in generating recommendations for improving the methodology for studying sustainability, and map a research agenda for future work. In consultation with our funding agency, we invited a subset of those participating in prior phases of the project, again selecting those who could represent research, healthcare leader, and funder roles in medicine, mental health, and public health. We again sought to maximize variation in gender, race, and experience level. We also explored potential to travel on the conference dates, replacing individuals who could not travel with those who were available, and chose only one member of any team. Participation was limited to 35 individuals to provide an in-depth working meeting; no single participant knew everyone else, and most did not know more than one or two other attendees. Each attendee nominated a key article or report they had authored, which were distributed to all participants prior to the meeting. Most attendees had participated in the CM, although we did not know who or their degree of participation. CM results were presented to jump start discussion. A panel of research and program funders responded to the “go zone”, or most important and challenging, items.

### Phase four: generating and consolidating recommendations

Working in small groups, conference participants were charged with “action planning” or generating recommendations from the CM results. Each group reported draft recommendations for discussion and refinement by the entire group on the second day. Within 10 days of the conference, each group leader prepared written summaries of their group’s recommendations, with detailed notes taken during the groups. The project leaders then refined the recommendations—conceptual, methodological, and related to research and healthcare infrastructure—for advancing sustainability research.

## Results

**Fig. 1:**
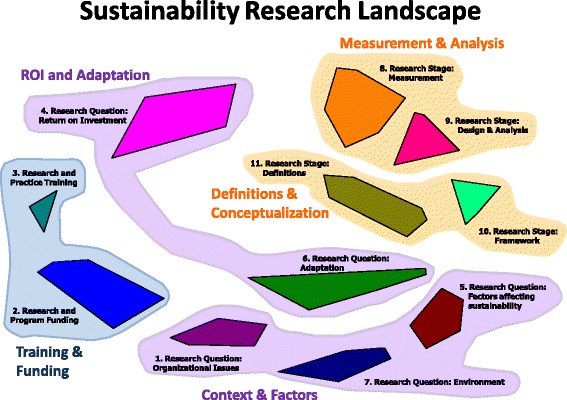
Sustainability Research Landscape

The CM process generated 11 clusters of ideas, labeled by the core team as the “Sustainability Research Landscape” (see Fig. 1). Table 1 presents the items and item loadings associated with each cluster. A CM’s stress value reflects the discrepancy between the original sorting data input by the users and the distances on the two-dimensional point map. Our map’s stress value of 0.32719 after six iterations fits well within the range established in other CM research and indicates that our map has good fit to industry standards [30].

**Table 1:** Statements by cluster by bridging

Cluster 1: RQ: organizational issues								
83	Paying special attention to issues related to the organizational learning curve and other issues related to evolution or drift of the practices							.41
77	Understanding when innovation or the natural life cycle of a program takes precedence over sustainability							.43
34	Identifying organizational adopter/sustainer archetypes							.45
30	Considering the organization as a learning organization rather than sustainability as an end-point							.52
76	Developing guidance for how organizations should decide to end a program and instead adopt a newer/better/more effective one							.69
5	Understanding the “rapid learning” or problem solving skills needed by key individuals/leaders and organizations in order to respond to changing environmental challenges							.78
	Count:	6	Std. Dev.:	0.14	Minimum	.041	Average:	.55
			Variance:	0.02	Maximum	.078	Median:	0.49
Cluster 2: research and program funding								
70	Funding							.09
67	Finding funding sources for conducting sustainability research							.22
86	Understanding and commitment on the part of funders for such work							.46
82	The availability of RFAs that explicitly call for sustainability research							.48
6	Determining why implementing agencies do/do not seek continued funding							.51
19	Fully exploring the role of adequate funding in sustainability							.62
41	Funds available to sustain a program							.70
36	Making sure that the research addresses required infrastructure and a viable business model to provide longstanding revenue support for the program							.73
	Count:	8	Std. Dev.:	0.21	Minimum	0.09	Average:	.47
			Variance:	0.04	Maximum	0.73	Median:	0.49
Cluster 3: research practice and training								
22	Establishing training in sustainability research							.20
15	Developing and implementing graduate curriculum relevant for sustainability researchers							.29
56	Training and capacity building in a public health agency							.42
91	Training and technical support to providers/deliverers of the program/intervention							.49
45	Identifying a broad set of journals and professional conferences that are good places for dissemination of sustainability research							.68
	Count:	5	Std. Dev.:	0.16	Minimum	0.20	Average:	.42
			Variance:	0.03	Maximum	0.68	Median:	0.42
Cluster 4: research question: ROI								
78	Determining the return-on-investment of sustainability given a variety of likely parameters (e.g., number of individuals reached; number of infections averted)							.43
50	Conducting ROI (return on investment) studies to make it clear to stakeholders and funders how much is actually gained when effective programs are sustained							.43
33	Determining the return-on-investment of sustainability during different time periods (e.g., 6-months, 12-months, 24-months)							.46
9	Investigating to what extent benefits (e.g. cost-savings, improved clinical outcomes) are sustained along with sustained use/behavior							.47
84	Conducting longitudinal cost-benefit analysis comparing implementation *vs.* sustainability							.50
46	Considering cost and economic issues from multiple perspectives							.66
23	Taking advantage of research already conducted in other fields and industries regarding sustained implementation of technologies and practices							.75
74	Creating survey of disciplines most involved in sustainability research							1.00
	Count:	8	Std. Dev.:	0.19	Minimum	0.43	Average:	.59
			Variance:	0.04	Maximum	1.00	Median:	0.48
Cluster 5: research question: factors affecting sustainability								
13	Figuring out how to predict sustainability based on the experience or knowledge of context gained through implementation							.23
39	Identifying key features of Evidenced Based Practice associated with variations in sustainability (e.g., strength of evidence; consistency with established practice; size of difference between prior and the new/evidence-based practices; staffing/other)							.25
53	Understanding which variables and factors are more important for sustainability than others							.25
7	Identifying key or core program sustainability components							.25
75	Investigating the relationship between sustained use, routinization and resistance to change							.29
12	Exploring whether the factors influencing sustainability differ from those influencing implementation							.30
60	Understanding if certain types of programs are less likely to be sustained							.30
69	Identifying common and independent factors that drive adoption *vs.* initial implementation *vs.* long-term use							.35
42	Exploring the supporting interventions (e.g., feedback) that are needed to sustain behaviors/use, for how long, and what intensity							.35
88	Understanding the reasons why strategies are/are not sustained							.39
	Count:	10	Std. Dev.:	0.05	Minimum	0.23	Average:	.30
			Variance:	0.00	Maximum	0.39	Median:	0.30
Cluster 6: research question: adaptation								
64	Understanding the tension between fidelity and adaptation as it pertains to sustained use or feasibility of continued use							.13
21	Documenting adaptations and their impact on the effectiveness of evidence-based practices							.14
47	Determining the core *vs*. peripheral or adaptable components of interventions							.14
55	Figuring out how to characterize adaptations							.16
54	Determining the point at which the intervention or program can no longer be considered sustained because of extensive adaptations							.18
66	Considering how the evidence-based program may change over time							.19
38	Understanding the implications of “partial” sustainability and adaptations							.24
28	Identifying interventions that are effective and cost-effective in fostering sustainability							.33
4	Framing sustainability as a partnership in which participants continue to adapt an intervention in response to changing conditions, while trying to remain true to its core principles							.34
	Count:	9	Std. Dev.:	0.08	Minimum	0.13	Average:	.21
			Variance:	0.01	Maximum	0.34	Median:	0.18
Cluster 7: research question: environment								
17	Defining the key attributes of organizations and systems that successfully sustain effective practice (e.g., ongoing leadership attention, ongoing measurement, systematic hardwiring of effective innovation, etc.)							.20
49	Identifying the key contextual factors (e.g., organizational characteristics) associated with variations in sustainability							.21
58	Defining and assessing the multiplicity of environmental variables that are likely to affect sustainability							.26
51	Discerning situations in which sustained use may be at odds with adopters’ (e.g., organizations) best interests							.31
8	Characterizing the context or environment of the intervention to be sustained							.33
65	Understanding cultural barriers to adoption							.33
62	Identifying which factors that advance or inhibit sustainability are amenable to management intervention							.53
63	Understanding how to sustain programs/policies in low resource settings							.71
	Count:	8	Std. Dev.:	0.16	Minimum	0.20	Average:	.36
			Variance:	0.03	Maximum	0.71	Median:	0.32
Cluster 8: research stage: measurement								
2	Developing methods for studying sustainability across the complexity dimension (e.g., sustainability of a specific clinical treatment *vs*. sustainability of a complex state-level chronic disease program)							.00
3	Using multilevel measurement							.00
37	Developing measures of sustainability (overall and sub-dimensions)							.01
18	Determining which analytic methods are most appropriate for sustainability research							.02
48	Identifying appropriate study designs for measuring sustainability							.04
85	Considering the role of self-reported data in assessing sustainability outcomes							.09
68	Identifying valid data sources for assessing sustainability							.10
81	Deciding how long to follow-up on a newly implemented program to determine whether it has been sustained							.10
27	Developing and validating fidelity measures for assessing adherence of a program to an evidence-based model							.11
89	Constructing reliable and validated tools to measure core sustainability constructs							.13
61	Having an operational definition of sustainability, with measurable criteria							.14
44	Determining “what” should measured as an outcome (patient level outcomes? fidelity? program activities? capacity?)							.26
14	The ability to move rapidly and use innovative research methods to learn from emerging opportunities and examples							.29
	Count:	13	Std. Dev.:	0.09	Minimum	0.00	Average:	.10
			Variance:	0.01	Maximum	0.29	Median:	0.10
Cluster 9: research stage: design and analysis								
72	Discussing how systems science methods such as modeling and network analysis can be used to study important sustainability questions							.03
59	Increasing use of non-experimental study designs in sustainability research							.08
43	Using a participatory approach to research that values the perspective of developers of original idea as well as the target group							.14
35	Clearly defining research hypotheses and goals linked to real-world application outcomes							.19
87	Identifying the indicators of sustained use so that we will know it when we see it							.25
29	Developing ways researchers can better integrate their documentation needs into the agency, so that it creates a smaller burden on those who deliver care (e.g., integrated data collection with electronic records)							.39
90	Conducting observational research of implemented programs to identify barriers/facilitators to sustainability							.42
	Count:	7	Std. Dev.:	0.14	Minimum	0.03	Average:	.21
			Variance:	0.02	Maximum	0.42	Median:	0.19
Cluster 10: research stage: frameworks								
11	Discussing whether to study sustainability separately from implementation (e.g., How are the two related? not related?)							.28
10	Testing of theories/frameworks for sustainability							.40
31	Creating greater distinction between predictors of sustainability (e.g., organizational capacity) and sustainability outcomes (sustained programming)							.42
26	Studying a greater variety of sustained activities, including interventions, programs, and policies							.43
16	Assessing the dynamic processes underlying sustained use							.50
57	Developing case studies to identify key characteristics of those that do sustain *vs*. do not							.63
	Count:	6	Std. Dev.:	0.11	Minimum	0.28	Average:	.44
			Variance:	0.01	Maximum	0.63	Median:	0.42
Cluster 11: research stage: definitions								
24	Conceptualizing and defining “sustainability”, its sub-dimensions, and related concepts (e.g., fidelity, routinization, institutionalization)							.08
79	Defining sustainability outcomes							.10
73	Creating a visual depiction of a sustainability model							.16
32	Identifying dimensions and degrees of sustained use							.16
80	Clarifying terminology (e.g., assimilation, institutionalization, continued use)							.17
71	Developing a formal conceptual model that links dissemination, implementation, and sustainability							.17
40	Balancing the use of pertinent theory, evidence, and experience							.29
20	Determining whether there is a standard level of initial “success” necessary before a project or organization or community is “eligible” to be considered for a “sustainability” evaluation							.32
	Count:	8	Std. Dev.:	0.08	Minimum	0.08	Average:	.18
			Variance:	0.01	Maximum	0.32	Median:	0.17

Ten statements were rated as both high in importance and high in challenge (Table 2). Of all 91 statements in our concept map, the question rated as most important was: *What factors are associated with sustainability*? The statement rated as most challenging for e sustainability research was: *What are the core principles that underlie the dynamic processes underlying sustained use?* Drawing on workgroup summaries, the core team consolidated the recommendations into three large domains: substantive questions for a sustainability research agenda, research methodology, and research infrastructure.

**Table 2:** Go zone: statements highest in importance and challenge in advancing research on sustainability

Domain one: research agenda
Defining the key attributes of orgs and systems that successfully sustain effective practice (e.g., ongoing leadership attention, ongoing measurement, systematic hardwiring of effective innovation, etc.)^a^
Assessing the dynamic processes underlying sustained use^b^
Testing of theories/frameworks for sustainability
Identifying common and independent factors that drive adoption *vs*. initial implementation *vs*. long-term use
Conducting ROI (return on investment) studies to make it clear to stakeholders and funders how much is actually gained when effective programs are sustained
Understanding how to sustain programs/policies in low resource settings
Identifying key or core program sustainability components
Determining the return-on-investment of sustainability given a variety of likely parameters (e.g., number of individuals reached; number of infections averted)
Identifying the key contextual factors (e.g., organizational characteristics) associated with variations in sustainability
Domain two: advancing methodology for sustainability research
Constructing reliable and validated tools to measure core sustainability constructs

^a^Most important of 91 statements

^b^Most challenging of 91 statements

### Domain 1: a substantive research agenda on sustainability

Five topics and questions were identified as important for research.

#### Improve clarity on the concepts and terminology used in sustainability research

The published literature reflects a wide range of different terms for sustainability, including fidelity, routinization, institutionalization, assimilation, durability, and continued use. Rarely are these terms defined conceptually or operationally. Participants recommended that all authors provide both conceptual and operational definitions of how they use the term sustainability. Definitions should align with previously published definitions or authors should provide a rationale for a new term or definition.

#### Identify the value of sustainability

One of the most important questions for empirical study is, “*what is the benefit, or value, of sustainability?*” This topic fell into the “go zone” of both important and challenging. Sustaining an intervention’s use is widely assumed valuable, in part to reap a return on the investments in its development, testing, and training. Yet, there is very little evidence of sustainability’s economic value. Research is needed to test whether, and to what extent, sustained use of an intervention contributes to service efficiency and effectiveness. For example, does sustainability contribute to greater population reach of evidence-based care and improved population health? Such questions, perhaps addressed through in-depth case studies, were viewed as important to funders, communities, intervention developers, and adopting organizations. The paucity of current evidence about sustainability’s value led this topic to be ranked as high priority.

#### Test the effect of interventions properties and adaptation on sustainability

While diffusion of innovation theory posits that intervention properties affect implementation [31], little research has tested this relationship or that between intervention properties and sustained use. We also do not know how treatment adaptations, subtle or dramatic, affect sustainability. As an example, participants noted that sustained use of film imaging for breast cancer detection was viewed as important until the advent of digital mammography, a dramatic adaptation with clear advantage over the earlier version of detection. Subsequently, few would regard sustained use of film imaging as desirable. Treatment adaptations and evolutions are widespread but varied in form, purpose, and effect. Thus, the field needs conceptual and empirical work on various treatment adaptations in relation to sustainability [32].

#### Test theories, frameworks, and models for their ability to explain and predict sustainability

A fourth priority for sustainability research is developing and testing models of intervention sustainability. A key question is whether dissemination, implementation, and sustainability have different predictors and thus whether their study requires distinct frameworks [17]. This work might begin with tests of implementation conceptual models and assessing the relative contributions of their constructs to sustainability or de-adoption over time. Empirically, work on sustainability conceptual models fell into the go zone of very important and yet very challenging.

#### Identify contextual factors that affect sustainability and understand explanatory mechanisms

A related priority is understanding the relationship between the service system context and sustainability. Context was understood broadly, including individual, inner setting, and outer setting factors as suggested by the Consolidated Framework for Implementation Research (CFIR) [33], such as leadership, organizational climate and culture, and system/policy factors [17, 21]. Particularly important is the identification of contextual factors associated with (1) implementation AND sustainability and (2) sustainability but DISTINCT FROM initial adoption. Ultimately, the field needs evidence-based management strategies to sustain effective interventions. Particularly important is learning how to sustain interventions in low resource health settings and how to plan/design for sustainability. Participants emphasized the importance of understanding role of funding, unfortunately rarely measured in published sustainability research [17] although identified as a reason for intervention de-adoption [34]. Finally, we need to understand whether factors associated with sustainability differ across populations, settings, problems, and interventions.

### Domain 2: advancing methods for sustainability research

Participants identified several methodological challenges in current sustainability research and corresponding recommendations.

#### Advance measurement

Consistent with the earlier priority on definitional harmony, the underdeveloped state of measurement poses one of the most serious methodological challenges. Participants agreed that confusion presently surrounds both the indicators and outcomes of sustainability. Participants recommended that sustainability researchers consistently employ these phrases:

- *Determinants of sustainability,* in reference to correlates and predictors of sustainability (organizational, contextual, and strategies); and
- *Outcomes of sustainability or sustainability outcomes* in reference to subsequent impact (healthcare improvement or public health outcomes) of sustained intervention use.

Sustainability research needs more measurement tools—more rigorous tools that are more consistently used. The field needs systematic reviews of measures currently used and published bibliographies and web-based repository of measures for sustainability determinants and outcomes. Measures work should be conducted to advance their reliability and validity. Moreover, an article should overview the varying thresholds reflected in extant research and recommend thresholds that constitute “sustained” use. Sustainability research needs specific reporting standards that include concept definition and measurement details. These recommendations are consistent with broader calls to advance harmonization of implementation science measures, an initiative three conference participants are involved in [35]. Another identified measurement priority is specification of level and unit of analysis within sustainability determinants and outcomes. Both determinants and outcomes can be measured at the individual provider, patient, organizational, health system, or community level. Studies should specify the unit of analysis and provide a rationale for its choice.

#### Locate and leverage appropriate sources of data on sustainability

The concept map and conference discussions reflected the challenge of finding data for measuring health intervention sustainability, exploring factors associated with sustainability, and prospectively testing strategies designed to enhance sustainability. New experiments and prospective studies require primary data collection, existing data. Yet data collected for other purposes may be leveraged for sustainability research, such as that from (1) health delivery sites that monitor quality by examining data on intervention delivery; (2) state health departments that monitor delivery of evidence-based interventions and policies; (3) completed sites of intervention randomized control trials and implementation studies, and (4) records of treatment or intervention dissemination and implementation purveyors, which typically collect detailed and dated information on intervention uptake, fidelity, and sustainability. Such data likely reflect intervention sustainment [16] and adaptation over time.

Secondary data are likely to vary in feasibility and quality. For example, procedure codes in medical records vary in specificity and hence in their usefulness for measuring sustainability. Secondary data also vary in cost and feasibility, and their use will require partnerships between sustainability researchers and the entities maintaining the data. Conference participants expressed concern that reviewers might regard previously collected data as old or out of date when, in fact, they might be optimal for sustainability research. Similarly, participants voiced concern that grant reviewers and funders favor new intervention development over sustainability research, although the latter should be viewed as high priority discovery science. Glasgow and Chambers [4] address the typical mismatch between how science is constructed and the demands of health and healthcare problems.

Conference discussions generated four characteristics of optimal data for sustainability research: (1) mixed in type, including both quantitative (e.g., procedure cores, dates of training, and intervention deployment) and qualitative data (e.g., case study narratives of sustainable and unsustainable scenarios); (2) reflecting perspectives of multiple stakeholders invested in sustainability (e.g. administrators, providers, patients or clients, frontline support staff, and treatment/procedure developers and researchers); (3) capturing variables at multiple levels of health delivery, including the organization, the intervention or program, provider behavior, and patient and system outcomes; and (4) collected over multiple time points, especially before an intervention is introduced and long enough afterwards to meet the threshold of “sustained”. Other recommendations include methods to cross validate self-reported sustainability with indicators in administrative data (e.g., retention of program-specific staff, procedure codes reflecting delivery, and inclusion of intervention costs in operating budgets).

#### Determine optimal designs for studying sustainability

Sustainability research requires designs for multiple, and relatively long, observation periods. Retrospective, observational, and prospective data are valuable in capturing intervention use beyond initial adoption. Outcome feedback loops may inform users’ decisions to continue use of an intervention, but time-to-intervention effect varies across various health conditions (e.g., infections respond more quickly to appropriate interventions than do episodic and chronic illness), and thus appropriate observation periods will vary as well. Extant studies employ widely varying observation periods, ranging from 1 [9] to 6 or 8 years [5, 36], with around 2 years the most common period [7, 8, 12, 22].

Conference participants recommended rigorous comparison of trend, panel, cohort designs to determine their advantages and limitations, as well as consideration of how sustainability research can leverage previously or continually collected data. Presuming long enough data collection, the relationship between adaptation and sustainability can be examined in comparative effectiveness and two-arm intervention trials that enable comparing an intervention delivered with strict fidelity to one continually modified. Multi-site trials of a single intervention in multiple sites can reveal how context influences intervention sustainment. A priority recommendation is the publication of a paper detailing design issues and recommended designs for sustainability research.

#### Use appropriate and robust analytic approaches

Data analysis constitutes a third challenge for sustainability research. The CM and conference discussions yielded two main recommendations: (1) sustainability research requires non-linear, longitudinal analysis, and (2) sustainability research should explore the utility of system science methods, particularly computational modeling and network analysis. Consistent with Rogers’ proposed S-curve model of innovation adoption, sustainability patterns are almost certainly curvilinear [31]. At any point in time, most health organizations are engaged in processes of beginning use of a new intervention while continuing to use another intervention and simultaneously phasing out others. Time series analysis, survival analysis, and nonlinear methods that capture feedback loops such as simulation and systems mapping, dynamic modeling, and network analysis are particularly appropriate for sustainability research. Given the large number of variables in most conceptual models of sustainability and the exploratory nature of much implementation research, mediational analyses are often needed.

### Domain 3: advancing the capacity, culture, and mechanisms for sustainability research

Several CM statements reflected participant concerns about the field’s capacity for sustainability research, stimulating discussion and recommendations around four aspects of infrastructure.

#### Capacity: develop a field of researchers well trained for sustainability science

Meeting participants—comprising both early stage investigators and leaders—agreed that most researchers are not prepared for the challenges of sustainability research. Participants generated a number of specific recommendations for preparing sustainability researchers, particularly for the methodological challenges noted above. Funders should: (1) prioritize and support career awards targeted to sustainability research; (2) incentivize junior-senior researcher partnerships in various grant mechanisms or through R01 supplements; and (3) provide or sponsor training in the distinct challenges of sustainability research, specifically measurement, design, and partnerships. Training programs in dissemination and implementation science and systems science should address sustainability research.

#### Capacity: train the healthcare workforce to sustain evidence-based care

The CM revealed concerns about the capacity of healthcare practitioners and administrators to lead their organizations through the challenges of sustaining evidence-based interventions. Conference participants recommended that foundations, universities, and professional associations provide better training for practice leaders and frontline providers in strategies for introducing, implementing, and sustaining evidence-based practices. These strategies should, of course, be evidence based and thus require a solid body of empirical work to identify ways to sustain evidence-based practices.

#### Improve the culture for sustainability research

The two day meeting was marked by widespread concern that the prevailing NIH culture of discovery does not align well with a priority on achieving maximum health impact. Even seasoned NIH reviewers and established investigators worried that sustainability is not viewed as an innovative scientific topic. Moreover, they expressed fear that secondary data, while ideal for some sustainability questions, may be viewed as less innovative than new data.

Accordingly, participants generated several recommendations. First, researchers should make and disseminate a data-driven case for sustainability as a return on investment in basic and clinical research. This case requires systematic reviews of existing data or proof of concept analyses using data simulation. Second, NIH wide workgroups should prioritize sustainability research, given that sustaining high-quality care is important across diseases, healthcare settings, and service delivery sector. Third, the dissemination and implementation research community should more forcefully prioritize research on the most challenging among implementation science issues, including scale up and sustainability. Sustainability research should be emphasized in grant program announcements and in panels at scientific conferences on advancing the science of dissemination and implementation research. Editors should be encouraged to publish sustainability research, perhaps in special journal issues or in a newly established annual review series within dissemination and implementation science. Fourth, sustainability champions—groups or individuals—should actively promote the prioritization and support of sustainability research. Champions could include the Robert Wood Johnson Foundation, the Surgeon General’s Office, Center for Healthcare Innovation, Institute of Medicine, and research funding bodies, especially the new PCORI and AHRQ, which sponsored this project. Finally, transdisciplinary and stakeholder-converged work should be encouraged and supported. Partnerships are needed among communities, healthcare organizations, frontline providers and support staff, intervention developers, funding organizations, and implementation researchers. One workgroup recommended creation of a virtual college or practice community of inquiry around sustainability, as through the Institute for Health Improvement.

#### Mechanisms to fund sustainability research

A final set of recommendations responded to concerns about how to fund sustainability research. Participants recommended that funding agencies issue grant requests for applications (RFA’s) specific to sustainability through: (1) supplements to implementation research and comparative effectiveness grants, in order to leverage opportunities to learn more about sustainability; (2) developmental (R21, R32) and R01 grants to identify and test strategies to sustain evidence-based interventions; (3) multi-year funding for data collection long enough to capture sustainability trends, including as needed activity drops (e.g., 1 or 2 years in the middle of a grant) and resumption of data collection after sufficient time to capture sustainability data; (4) rapid start up grants for natural experiments in sustainability; and (5) funds to capture research-practice handoffs phases of intervention research, for purposes of observing how newly implemented interventions are implemented in a naturally occurring healthcare, and if and how they are sustained. Budgets should be sufficient to cover the costs associated with mixed methods, systems science, and longitudinal approaches. Implementation science and sustainability research especially need grants for methods and infrastructure development, including conference grants. Finally, concern emerged about grant review quality. Participants underscored the importance of ensuring that review panels possess expertise in the unique substantive and methodological features of sustainability research such as detailed here and encouraged the inclusion of appropriate community and health practice stakeholders. In particular, review committees should include experts in longitudinal design and analysis, systems science, and multi-level analysis.

### Evaluation

We administered and collected an evaluation instrument before participants left the meeting. The conference was rated very positively. On a 1–5 scale, item means ranged from 4 to 5. Several participants urged a follow-up meeting and action that would extend beyond the 1-year funding period.

## Conclusions

Despite the annual investment of tens of billions of U.S. tax dollars on health research and progress in developing, testing, and implementing evidence-based healthcare, we have limited understanding of how to sustain quality health care in routine services. Papers commissioned for the 2010 AHRQ supported Conference to Advance the State of the Science and Practice on Scale-up and Spread of Effective Health Programs identified the lack of attention to sustainability as a major challenge to subsequent scale-up [37].

To advance knowledge of sustainability, this project brought together thought leaders and researchers invested in research on sustaining evidence-based health. Scheirer and Dearing’s conjecture [20] that even researchers focused on a topic as specific as sustainability often are unfamiliar with work done outside their own area was validated as most conference attendees did not know more than one or two other participants.

Our approach had strengths and limitations. Participation rates for the time-consuming and novel (for many) CM were less than we hoped, with rates for sorting, importance ranking, and challenge ranking even lower than for the brainstorming activity. This may have limited the breadth of perspective brought to the task. However, given the heterogeneity of participants’ roles and backgrounds, the strong consensus around research priorities and imperatives was striking. Moreover, our data’s properties fit industry standards well and generated rich debate over the work to generate recommendations.

Recommendations included: use of clearer concepts and terms in reference to sustainability; greater reliance on theory and conceptual models to frame study questions; more operational definitions with clear measurement guidelines for sustainability thresholds; and more robust designs and analytic methods for testing the relative contribution of explanatory factors. Building on Stirman’s [17] critique of the confused and underdeveloped state of definitions in sustainability research, participants concurred that sustainability researchers should distinguish their work as focused on the outcomes of sustainability—including the duration, maintenance, or continued delivery of an evidence-based healthcare intervention and their organizational and public health impacts; or the determinants of sustainability—such strategies as training, management and financial practices, and organizational and contextual factors that can help achieve sustainability. Participants ranked high in priority the recommendation that sustainability researchers explicitly test conceptual models, with an aim of discovering the applicability of adoption and implementation models or the necessity of distinct models of sustainability.

Participants expressed concern that the prevailing emphasis on basic and clinical discovery—particularly in the newly created National Center for Advancing Translational Sciences—jeopardizes the science required to inform long-range improvements in the nation’s public health. Such science demands better understanding of how to sustain evidence-supported interventions, particularly in under-resourced settings that serve vulnerable populations. For too long, sustainability has been a concern only at the tail end of the research pipeline [38] and thus insufficiently addressed. Continued efforts are needed to bridge these pockets of work, consolidate what is known, identify unanswered questions, and formulate a plan for accelerating an empirical base for sustaining healthcare improvements.

### Protection of human subjects

The Washington University Human Research Protections Office approved this study. Participants involved in the brainstorming, sorting, and ranking had to provide online consent prior to participating in these three phases of CM.

## Abbreviation

CM: Concept mapping
